# Indigenous LGBTIQSB + People’s Experiences of Family Violence in Australia

**DOI:** 10.1007/s10896-023-00539-1

**Published:** 2023-03-30

**Authors:** Karen Soldatic, Corrinne T. Sullivan, Linda Briskman, John Leha, William Trewlynn, Kim Spurway

**Affiliations:** 1 Institute for Culture & Society, Western Sydney University, Parramatta, NSW Australia; 2 School of Social Sciences, Western Sydney University, Parramatta, Marrickville, NSW Australia; 3 School of Social Sciences & Psychology, William Trewlynn InCulture, Sydney, NSW Australia; 4 NSW Child, Family & Community Peak Aboriginal Corporation, AbSec, Marrickville, NSW Australia; 5 InCulture, Sydney, NSW Australia

**Keywords:** Indigenous, First Nations, Aboriginal, Torres Strait Islander, LGBTIQ+, Australia, Family Violence

## Abstract

**Purpose:**

This article uses an Indigenous concept of family violence as a frame to interrogate interviews held with Indigenous LGBTIQSB + people in Australia. The article reorients family violence away from Western heteronormative framings and aims to contribute towards a new conversation about family violence.

**Methods:**

A qualitative thematic analysis was used to analyse 16 interviews with Indigenous LGBTIQSB + people in the state of New South Wales, Australia. This is one of a series of articles that provide preliminary findings from a research project into the social and emotional wellbeing of Indigenous LGBTIQSB + young people living in New South Wales.

**Results:**

The interviews highlight the complex impact family violence on Indigenous LGBTIQSB + youth. The article shows differences in reactions between family and community in urban settings with those experienced in rural settings highlighting intergenerational differences, with older family members such as grandparents, more likely to exhibit negative reactions and behaviours. These experiences are interconnected as many young people were living in urban areas while extended family often lived in rural or remote communities.

**Conclusions:**

The findings of this study demonstrate the intersectional nature of family violence highlighting the fact that Indigenous LGBTIQSB + young people are integral parts of extended kinship networks, families and communities and are deeply impacted by any acts of family violence. The study’s findings also support current research into family and community violence for LGBTIQ + people that shows the differential behaviours and actions of rural and urban families as well as the different reactions between generations within families.

## Introduction

To the best of our knowledge, there is nothing written about the experiences of family violence by Indigenous LGBTIQSB+ (Lesbian, Gay, Bisexual, Trans, Intersex, Queer, Sistergirl, Brotherboy plus)^1^ people living in the country now known as Australia. In a recent systematic review of the evidence, for example, Carlson et al. (2021) did not find any publicly available evidence on the rates of family violence for Indigenous LGBTIQSB + people in Australia. No studies to date have taken a family violence perspective to the lived experiences and aspirations of Indigenous LGBTIQ + people in Australia and even though there is some tangential evidence about family violence in the literature or even topics such as intimate partner violence for Indigenous LGBTIQ + people despite their ostensible importance (see Briskman et al., 2022; Dudgeon et al., 2015; Hill et al., 2022a, 2022b; Kerry, 2014, 2018; Liddelow-Hunt et al., 2021, 2023; Riggs & Toone, 2017; Soldatic et al., 2021a, 2021b for example).

Indeed, until very recently it was challenging to find any information about Indigenous LGBTIQSB + people at all. Previous evidence syntheses found only one extant study that addressed Indigenous LGBTIQSB + social and emotional wellbeing in Australia (Soldatic et al., 2021a; Spurway et al., 2020). This has changed, however, with more papers on this topic papers being published in the last two years although none directly address Indigenous LGBTIQSB + people’s experiences of family violence (see for example, Briskman et al., 2022; Day, 2020; Hill et al., 2021; Liddelow-Hunt et al., 2021; Soldatic et al., 2021a, 2021b; Spurway et al., 2022; Sullivan et al., 2022a, 2022b, 2022c; Uink et al., 2020).

Evidence about Indigenous communities and non-Indigenous LGBTIQ + populations, however, suggests that family violence could be relatively high for Indigenous gender and sexuality diverse people (Carlson et al., 2021). Both Indigenous and LGBTIQ+ (Lesbian, Gay, Bisexual, Trans, Intersex, Queer plus) people experience report higher rates of social, emotional, physical, and sexual violence than equivalent groups in Australia (ABS, 2019). Indigenous people, for example, were hospitalized 32 more times for family violence than non-Indigenous Australians in 2016–17 (AIHW, 2019). At the same time, people who identify as LGBTIQ + in Australia are two to three times more likely to report physical, social, emotional, and sexual violence than their heterosexual counterparts (Ovenden et al., 2019: Szalacha et al., 2017). Family violence in both cases is also highly gendered. More Indigenous women reported family violence than men (AIHW, 2019) and LGBTIQ + women reported more abuse than LGBTIQ + men in similar surveys (Ovenden et al., 2019: Szalacha et al., 2017). The intersection of how Indigenous and LGBTIQSB + affects people’ experiences is not currently evident, but physical, emotional, and social harm of this kind, “not only has a negative impact on the physical and mental health of the victim, but it can also affect family members, friends and the broader community” (AIHW, 2015, p.44).

The connected and relational nature of family violence is reflected in Indigenous framings of family violence. “Family violence” is the preferred term to “domestic violence” within Indigenous communities, reflecting a wholistic understanding that includes the social, physical, emotional, and sexual aspects of the phenomenon (AIHW, 2015; Carlson et al. 2021). For Indigenous people, violent behaviour within families and communities cannot be separated from broader structural violence caused by intergenerational trauma arising from punitive historic and contemporary colonial settler policies and processes. Carlson and colleagues (2021) argue that in Indigenous communities, “the precursor for family violence is colonisation” (p. 5). This is supported by the extant research, with families that endured the trauma, loss and grief associated with the Stolen Generations, for example, more than twice as likely to be victims of family violence than other Indigenous families (Carlson et al., 2021).

This definition of family violence recognises the fact that individuals do not exist in isolation, that they are part of extended kinship networks, families, and communities and that their experiences, behaviours and attitudes impact family and community relationships. Non-Indigenous framings, for example, tend to see illicit substance use as leading to domestic violence and the breakdown of family relationships (AIHW, 2015). However, an Indigenous framing would view structural violence, family violence and dysfunctional family relationships as the precursor to an individual’s use of illicit substances and potentially harmful behaviours. Consequently, family violence at its foundation is the cause of other forms of violence at individual, family, and community levels, with family violence expressed through behaviour within individuals, families, and communities. This framing of family violence is not limited to physical violence or verbal abuse but “is any use of force, be it physical or non-physical, which is aimed at controlling another family or community member and which undermines that person’s wellbeing” (Calma, cited in Carlson et al., 2021, p. 4). As such, family violence “includes not just physical, sexual, psychological, social and economic abuse but also activities such as family feuding, elder abuse, child abuse, and antisocial and aggressive behaviour by youth” (Carlson et al., 2021, p. 4).

In discussing family violence and Indigenous LGBTIQSB + people, it is also important to expand heteronormative understandings of genders, sexuality, and relationships. In reviewing the evidence on gender roles in Indigenous communities, the team at the Aboriginal Health Council of South Australia (AHCSA, 2019) did not find any primary research that focused on Indigenous LGBTIQSB + people in Australia. AHCSA (2019) also reported that the current literature reflected “rigid gender stereotypes” based solely on sexual characteristics (p. 5). This reflects conventional binary, heteronormative value systems and that the belief that family violence is predominantly about violence by heterosexual cismen against heterosexual ciswomen in intimate relationships (Carlson et al., 2021). It also reflects the ways in which the Australian settler state has (re)aligned gender roles within Indigenous family and kinship networks privileging non-Indigenous concepts of gender, sexuality, and family life (O’Sullivan, 2021).

The ways in which the Australian state and service providers construct ‘family’ as a heterosexual cisman, heterosexual ciswoman and their immediate children is illustrated in major policy interventions such as The Stolen Generations (HREOC, 1997). Here, Indigenous people were removed from their families and communities and given to non-Indigenous families to adopt; to use as unpaid domestic servants or placed in workhouses, boarding schools, Christian missions, and orphanages (HREOC, 1997). As part of a broader, long-term strategy aimed at extinguishing Indigenous sexualities and gendered roles (Sullivan, 2018), the Stolen Generation were subjected to heteronormative regulation and control by the settler state and service providers about who were deemed ‘appropriate’ sexual and intimate partners (HREOC, 1997).

The decentering of heteronormative framings of family violence is also an important theme found in the literature on non-Indigenous LGBTIQ + experiences of family violence. Lusby and colleagues (2022) report on how to improve family violence service outcomes for LGBTIQ + people shows that more than two fifths of this population experience family violence in some form but only 25.9% reported recent experiences of family violence to service providers and 5.9% to police. A significant barrier to finding support for LGBTIQ + people was the challenges most people surveyed found in naming and identifying behaviours “outside of typical framings that emphasise heterosexual and/or cisgender experiences” (Lusby et al., 2022, p. 4). Typically, LGBTIQ + people surveyed thought that only overtly physical or sexual violence constituted family violence downplaying other more subtle acts such as coercive or emotional control and psychological (Lusby et al., 2022). Campo and Tayton (2015) also found that, although LGBTIQ + people reported experiencing intimate partner violence at a similar rate to heterosexual cisgendered people, an “invisibility” about LGBTIQ + relationships was prevalent in service provision programming in Australia because many service providers lacked awareness of the complexities of LGBTIQ + relationships and act based on heteronormative and cisgendered assumptions about intimate relationships (Campo & Tayton, 2015). It is these kinds of assumptions about gender and sexuality diversity that can influence the ways in which family violence is conceptualized and framed that can create barriers to service provision and current framings of family violence.

Understanding the ways in which settler heteronormative values were forced upon Indigenous peoples in Australia is very important in terms of understanding how family violence is enacted. Indigenous sexualities and genders have the potential to disrupt “static conventions of performing gender and opens up new categories for naming sexuality, with the potential the potential to remove labelling altogether, and to unsettle power associated with gender and sexuality normativity” (Bayliss, 2015, p. 4). If we consider gender diversity as being dynamic and changeable, this creates the potential for “a set of possibilities for gender identity, embodiment and emotion that until recently have been unacknowledged in the Eurocentric Western imagination” (Sullivan & Day, 2019, p.2). However, Indigenous LGBTIQSB + genders and sexualities cannot be understood unless Indigenous voices are at the centre of any research project (Sullivan, 2021). As the most authentic sources of their own experiences and aspirations, giving voice to Indigenous people “supports the (re)construction of Indigenous representations on our own terms” and decenters it away from binary non-Indigenous heteronormative norms (Sullivan, 2021, p. 54). We hope to add to this discussion to expand current understandings of interpersonal relationships, families and communities, further decentering framings of family violence away from settler concepts based on non-Indigenous heterosexual, nuclear family groupings.

## Literature on Family Violence

Although there are no studies that directly investigated Indigenous LGBTIQSB + people’s experience of family violence in Australia, there is an emerging body of scholarship that tangentially discloses information on the topic. This literature shows that the types of violence reported in family and community was diverse, including phobic and racist behaviours, abuse and acts of physical and sexual assault. The literature also highlights differences within families, with older relatives and extended family often reported to be more likely to be LGBTIQSB + phobic than younger family members. This was found to be especially true of extended family and kinship networks in rural and remote Indigenous communities.

### Family Violence in Family and Community

Families and communities are not always safe places for Indigenous LGBTIQSB + young people. Sullivan et al.’s (2022a) participants, for example, experienced discrimination, a lack of acceptance, being disowned by family and rejected in community. One participant saying she felt she was not, “allowed to be different in the country”, referring to the people, lands, and waters she felt attached to as an Indigenous Australian (Sullivan et al., 2022a, p.12). Dudgeon et al. (2015) also found that Indigenous LGBTIQSB + people reported hostility within Indigenous communities and that they believed that this hostility not only impacted them directly but also diminished and fragmented overall community wellbeing. Dudgeon et al. (2015) also found that misunderstandings in addition to micro and macro aggressions against Indigenous LGBTIQSB + peoples compound trauma and shame for individuals and lead to negative outcomes for individuals, families and communities leading to problems such as drug misuse and collapse of communal trust and social capital.

This is also supported by research from other authors such as Hill et al. (2021), who found that nearly two thirds of their survey participants experienced interpersonal violence of some kind (physical, emotional, and financial harm perpetrated by another person) within their family circle (mother, partner, father, brother, uncle, sister) and/or close family friends or partners. Many Indigenous LGBTIQSB + youth also reported experiencing some form of family violence from more than one perpetrator (Hill et al., 2021). Nearly a quarter of survey participants, for example, said they were not accepted by Indigenous Elders or community leaders because of their gender and/or sexuality and that in Indigenous communities they felt “invisible” (Hill et al., 2021, p. 19).

Sullivan and colleagues (2022a) also found many of their survey participants reported experiencing family violence in their childhood homes, community spaces and households. One participant stating that, “Some comments that were made against even told me I should end my life” (quoted in Sullivan et al., 2022a, pp. 203–204). In a later paper, Sullivan et al. (2022b) reported that when a participant’s stepfather behaved negatively towards their child’s gender diversity, the participant’s mother told him, “The door is there. She’s not your child but you’ve raised her, and you’ve got to be there for her. What can we do? Sorry, but your child has more value over religion, so support her or get out” (Sullivan et al., 2022b, p. 13). Other studies support these accounts with Indigenous LGBTIQSB + people repeatedly reporting violence such as abuse, insults, ridicule, exclusion, isolation, physical assault, and rejection by family and community members (Dudgeon et al., 2015; Liddelow-Hunt et at., 2021; Riggs & Toone, 2017; Soldatic et al., 2021a, 2021b; Sullivan et al., 2022a, d).

Experiences with family and community can be particularly challenging for Sistergirls and Brotherboys. Riggs and Toone (2017) found that while some Sistergirls reported experiencing love and acceptance from some members of their families, others experienced threats of violence, hatred and rejection from families and friends because of their gender diversity. This placed some Sistergirls at risk of involuntary sex work, abuse, illicit drug and alcohol misuse and poor mental health, many leaving their communities to feel safe. Crystal Johnson (2015) spoke about their personal experiences of violence from family members in the Tiwi Islands and explains the impact this had on other family members: “My aunties used to bash my mum because I was a Sistergirl” (Johnson, 2015, p.4), and “My mother was bullied, and my father’s sisters used to spit at her. When she would see them and try to talk to them, they walked straight past her” (Johnson, 2015, p.5). Johnson (2015) also spoke about ‘payback’ on the Tiwi Islands, in which family members can be subjected to acts of community violence for having a Sistergirl in the family. Johnson’s elder brother, for example, was physically assaulted at his initiation in payback for Crystal’s gender identity and the family threatened not to say anything to police or doctors (Johnson, 2015). In another autobiographical piece, Clancy (2015), also speaks about their feelings of rejection as a transman, when they were not allowed to participate in Corrobborees with the boys but were made to stand at the back with the girls. This, Clancy (2015) felt, was a denial by community of their authentic selves. These firsthand stories are supported by scholars such as Kerry (2014, 2018), who also found that sistergirls experienced “social exclusion, discrimination (e.g., racism and transphobia), and lack of support networks” because of violent behaviours experienced within family and community (Kerry, 2014, p. 181).

Scholarship also highlighted the importance of violence enacted on Indigenous communities by colonisation and heteronormative Christian values and how this shaped attitudes towards gender and sexuality diversity within Indigenous communities (Hill et al., 2021; Liddelow-Hunt et al., 2021; Soldatic et al., 2021a, b; Sullivan, 2021; Sullivan et al., 2022a, d) argues, for example that western and Christian values about sexuality and/or gender influenced Indigenous communities who conflated these values with what they believed to be ‘traditional’ Indigenous cultures. This has had a significant impact on Indigenous communities, families, and individuals, “Due to the complex histories of colonisation, dispossession, Stolen Generations and other social determinants of health and well-being the prevalence of Interpersonal Violence is also much higher for Aboriginal and/or Torres Strait Islander people” (Hill et al., 2021, p. 17). The consequences of LBTIQSB+-phobia in Indigenous communities means that some gender and/or sexuality diverse people feel forced to leave their communities of origin, moving to cities where they can openly express their sexuality and/or gender (Sullivan, 2021).

### Racism in Non-Indigenous LGBTIQ + Communities

Indigenous LGBTIQSB + people can consider non-Indigenous LGBTIQ + communities as their ‘second family’ especially if they are escaping LGBTIQSB+-phobia in Indigenous families and communities and, as such, non-Indigenous LGBTIQ + spaces are one of the community spaces in which participants live and experience family violence (Soldatic et al., 2021b). A young gay cisman, for example, describes his discovery of the non-Indigenous LGBTIQ + community, “I found a whole new family and believe it or not, I think it’s so much better than what my old family is and was. And you can’t pick your family, but you can pick your friends. And I think as LGBT people, we get to choose our families anyway” (quoted in Soldatic et al., 2021b). Non-Indigenous LGBTIQ + communities were often seen as spaces where Indigenous LGBTIQSB + people could safely express their sexuality and/or gender.

However, a common experience of Indigenous LGBTIQSB + people was that of racism in the form of micro aggressions, discrimination and explicit verbal and physical abuse within non-Indigenous LGBTIQ + communities. Sullivan and colleagues (2022a, 2022d) described participants’ experiences of racism within these communities, which deterred them from going to bars, clubs and other LGBTIQ + spaces leading to increased feelings of isolation and exclusion. Racist abuse and threats were also reported in LGBTIQ + spaces and events as well as in online forums and social media. Clancy (2015), for example, describes an encounter with racism in an online trans Facebook community where two non-Indigenous trans group members accused Clancy of being ‘too white’ to be Indigenous. Hill and colleague’s (2021) survey participants also spoke about different types of explicit discrimination in the non-Indigenous LGBTIQ + community such as being rejected as a partner because of their Indigeneity or non-Indigenous people making explicitly racist comments. Sullivan (2021) found that their participant, Jack, reported that although their sexuality is accepted, their Indigeneity is not. As a sex worker, Jack could not advertise being an Indigenous Australian as he feared being subjected to racism in the LGBTIQ + community (Sullivan, 2021). Consequently, Jack feels both culturally excluded in the LGBTIQ + community and socially excluded in the Indigenous community.

### Failures in Service Provision

Although not specifically speaking about family violence services, this body of scholarship also highlights some key concerns for Indigenous LGBTIQSB + people with accessing services of various kinds. In terms of family violence, Dudgeon et al. (2015) argues for the need for culturally and gender/sexuality appropriate service provision that also recognises the impact and importance of white settler heteronormative colonial framings and the significant historical and contemporary trauma within Indigenous communities underlying collective and individual acts of violence. Liddelow-Hunt and colleagues (2021) also found that, in the case of mental health, service provision was inadequate and failed to provide appropriate levels of acceptance and support for Indigenous LGBTIQSB + young people. Liddelow-Hunt (2021) proposed that services recruit and retain more Indigenous LGBTIQSB + practitioners, that services improve their cultural competencies and understanding, as well as explicitly and openly demonstrating they are allies to Indigenous LGBTIQSB + youth.

The literature also revealed that there is a lack of robust research and useful information that could potentially assist services to design, create and run more culturally and gender/sexuality appropriate programs in education (Rhodes & Byrnes, 2021; Sullivan & Day, 2019), family violence (Carlson et al., 2021) sexual health (Sullivan et al., 2022a, b; Spurway et al., 2022) and social and emotional wellbeing (Briskman et al., 2022; Liddelow-Hunt et al., 2021; Spurway et al., 2022; Uink et al., 2020; Sullivan et al., 2022b) in discussing their study participants’ experiences of service provision highlighted, “Indigenous LGBTIQ + young participants discussed their lack of input in services, with service providers not seeking their consultation to help develop support that focuses on their inter-sectional identities” (p. 12).

### Lack of Disclosure of Family Violence in Indigenous Communities

This small set of studies is significant because they disclose instances of Indigenous LGBTIQSB + people reporting openly about family violence. Within the racist and heteronormative context of colonial settler service and welfare provision, many Indigenous people do not like to speak about negative experiences within family and community to outsiders (Carlson et al., 2021; Sullivan, 2020). Carlson et al. (2021) sums up some of the reasons why Indigenous people do not disclose family violence pointing out that the “reasons for non-disclosure are numerous and complex: shame; fear of reprisals from the perpetrator and others in the community; fear of the ramifications of involvement of the justice system and other government services; a perception that abuse is normal and something that has to be endured; the difficulty of keeping something private in a close-knit community; poverty and isolation; and a lack of culturally appropriate services” (p. 5). Sullivan (2021) identifies other reasons for the “silences” researchers might encounter in Indigenous communities, for example, who sets the research agenda, power differentials, knowledge ownership and skepticism from community gatekeepers about the aims of research and potential benefits for Indigenous communities.

Reporting family violence can have serious consequences given a history of highly racialised colonial settler state policies that led to catastrophic state led interventions. As part of their ongoing attempts to replace Indigenous populations, settler states continue to practice discriminatory policies that favour migrant settler populations over that of the Indigenous peoples and systematic attempts to extinguish their pre-existing claim to the colony’s lands and waters (Veracini, 2010). These kinds of policies can be demonstrated through one notorious case, The Stolen Generation. ‘The Stolen Generation’ was the systematic removal of Indigenous children from their allegedly ‘dysfunctional families’ resulting in intergenerational trauma and grief that still adversely affects every Indigenous community across Australia (HREOC, 1997). An Inquiry by the Australian Human Rights and Equal Opportunities Commission (HREOC) into the history of the ‘Stolen Generation’ found that Indigenous children were forcibly removed without evidence of neglect or abuse. In effect neglect was just assumed: “In contrast with the removal of non-Indigenous children, proof of ‘neglect’ was not always required before an Indigenous child could be removed. Their Aboriginality would suffice” (HREOC, 1997, p. 9).

## A note on Terminology

The intersection of Indigenous families and communities is complex as Indigenous families and communities do not follow non-Indigenous norms. Families and communities are close intertwined, involving interconnected relationships that are not solely based on close biological relationships non-Indigenous people would consider make up a ‘close family’ such as parents and siblings. For Indigenous people, family can also include non-biological community members who are not related to participants from a non-Indigenous perspective but are called ‘Aunties’ or ‘Uncles’ or ‘Cousins’ by Indigenous people, for example (Walter, 2017).

Colonial-settler and binary, heteronormative, heteropatriarchal, cisgendered values have meant that the identities, and hence nomenclature, of Indigenous LGBTIQSB + peoples, continues to change as they (re)connect with their cultures. In this paper, we use the terms “Indigenous” when speaking of Indigenous peoples globally but use country-appropriate terms when discussed in studies or by participants. Many of the participants also use the term, “Country” to describe the lands and waters they feel connected to: “Many Indigenous people derive their self-identity from the land and seas. In contemporary Indigenous Australia, Indigenous people often identify themselves as coming from their ancestral country, or ‘their’ place” (Burgess & Morrison, 2007, p. 180).

For Indigenous people living in Australia, our team prefers the term ‘Indigenous’ over other current terms such as ‘First Nations’ to represent the Aboriginal and/or Torres Strait Islander peoples of Australia. In general, we use the terms as set out in the literature we are describing or as used by our participants including Indigenous terms such as ‘mob’. For gender and sexuality diverse people, we use acronyms such as LGBTIQ + also dependent on their use used by participants or the studies analysed here. LGBTIQSB + is an Indigenous acronym unique to Australia and includes people who identify as Lesbian, Gay, Bisexual, Trans, Intersex, Queer, Sistergirl, and Brotherboy. We use an Indigenous understanding of the term ‘family violence’ to represent discord, discrimination, abuse, and violence reported in families and communities.

Given the absence of research into family violence and Indigenous LGBTIQSB + people in the country now known as Australia, there is little substantial evidence that can be drawn on to understand this issue in depth. This article will hopefully begin a conversation about what Indigenous LGBTIQSB + people reported experiencing in their families and communities. Indigenous LGBTIQSB + experiences of family violence will also broaden the current discussion on family violence in Indigenous families and communities and destabilize normative ideas about who is impacted by different kinds of family violence, especially with regards to gender and sexuality diversity. This will eventually lead not only to broader understanding but also innovative approaches and policies that can improve service provision but also the lives of Indigenous LGBTIQSB + young people.

## Method

### Participants & Recruitment

Using an Indigenous framing of family violence to analyze existing research on Indigenous LGBTIQSB + people in Australia, this article interrogates the findings of interviews with Indigenous LGBTIQSB + people and their experiences of family and community in the state of New South Wales (NSW) in the country now known as Australia. This article uses 16 interviews conducted between April 2020 and November 2021 with Indigenous LGBTIQSB + young people living in NSW aged 14–25 years. Almost all the participants transcripts used here are ciswomen or cismen. Unfortunately, no Sistergirls were successfully recruited for the project, however, the team recruited a Brotherboy and one trans/non-binary person.

Participants were recruited using Indigenous LGBTIQSB + social networks, radio, social media posts and service provider networks. Service providers who supported and assisted with recruitment included ACON, BlaQ Aboriginal Corporation, Twenty10 (Incorporating the Gay & Lesbian Counselling Service), Campbelltown City Council, and Infant Child Adolescent Mental Health (ICAMHS) in NSW Health. Participants attended either in person or online, interviews were approximately one hour in length. Prior to the Covid-19 pandemic, interviews were held as much as possible in person at accessible, safe Indigenous spaces such as the Kimberwalli Centre in Western Sydney. However, the Covid-19 pandemic and government restrictions in New South Wales in 2020–2021 meant that interviews were then held online.

Participants identified as members of the Birpai, Bundjalung, Djangadi, Gumbayngirr, Kamilaroi, Meriam, Murri, Muruwari, Mineng/Noongar, Nunukul, Wakka Wakka, Wiradjuri, Wuthathi and Yuin peoples. Many participants identified with more than one First Nations people with intersecting genders and sexualities including Bisexual, Brotherboy, Demisexual, Gay, Lesbian, Non-Binary, Queer and Trans. Participants all lived in urban areas, mostly located within the greater Sydney area, with one participant from a small regional urban center located in the northern tablelands of NSW.

### Research Design & Procedure

Each participant’s interview was thematically analysed to allow for concepts and themes to emerge. The analysis was inductive, that is, analysis started with pre-conceived concepts but also allowed themes to emerge during analysis (Charmaz, 2014). Analysis used different levels of open, axial, and selective coding as foundational techniques to interrogate the text (Charmaz, 2014; Strauss and Corbin, 2012). The research team read through the transcripts to open up the text and identify broad themes. Interview transcripts were then read line by line to further capture common themes and conceptual categories. Themes were organised into categories, identifying key relationships and linkages (Charmaz, 2014; Strauss and Corbin, 2012). Using an iterative approach, the themes and categories built on each other and generated higher levels of abstraction, which then informed later stages and generated increasingly meaningful and thick description.

Questions used in the interviews were codesigned by the research team in the early stages of the project in close consultation with ACON Aboriginal Project and BlaQ Aboriginal Corporation. Field research was conducted by researchers from both Western Sydney University and BlaQ Aboriginal Corporation. Preliminary data analysis was carried out by an experienced qualitative researcher from the WSU team with later analytic stages involving researchers from WSU and BlaQ Aboriginal Corporation. All authors had access to transcripts to double check analyses reflected information provided by participants.

This article was reviewed by Indigenous LGBTIQSB + people from the project’s NSW Indigenous Research Governance group, and the NSW Aboriginal Health and Medical Research Council’s (AH&MRC) Human Research Ethics Committee (HREC). The project received ethics approval from the AH&MRC (HREC Ref. 1536/19) on 27 August 2019 and approval to publish this article on 24th August 2022. All quotes have also been reviewed and approved for publication by each of the participants quoted in this article. This article is one of several outputs from a research project funded by the Australian National Health and Medical Research Council (NHMRC) under its Targeted Call 2018 Indigenous Social and Emotional Wellbeing Funding Round (Grant ID: 1,157,377). The larger NHMRC project aimed to improve understanding of young Indigenous LGBTIQSB + people’s wellbeing and their experiences and aspirations regarding service provision.

### Author Positionality

The authors acknowledge the importance of critically evaluating their positionality as this will impact on the analysis and interpretation of data collected in this project. However, best practice is founded on the idea that no author should be forced to disclose their identities if they do not want to. The authors feel that a general statement is more appropriate given the dominance of toxic heteronormativity, the need for the authors to feel safe, and to choose who they will disclose to and when. When the manuscript of this article was drafted, some authors identified as queer ciswomen or cismen, and three of the six authors were members of the Wiradjuri, Tingha, Birra Gubba, Wakka Wakka, Tongan, and Dunghutti peoples. The other three authors identified as Jewish, Istro-Romanian/Croatian and Anglo-Celtic Australians.

## Results

Family violence and related behaviours were reported in all 16 interviews. These behaviours and actions took place within intersectional spaces in which Indigenous LGBTIQSB + people live and work (Indigenous families and communities; non-Indigenous LGBTIQ + communities and non-Indigenous spaces). The most significant and most discussed was the impact on participants of phobic reactions in Indigenous families and communities. The second most common space was non-Indigenous LGBTIQ + communities. Family and community are important, as one participant put it, “we [Indigenous people] are relationship centred” and negative reactions of family and community caused participants a great deal of distress and discomfort sometimes forcing them to hide their LGBTIQSB + identities to feel safe. For some participants, this meant they could not return to Country to visit grandparents or community to avoid phobic behaviours. Unlike the non-Indigenous LGBTIQ + community where people experience intimate partner violence at similar rates to people who identify as heterosexual (Campo & Tayton, 2015), only three participants discussed experiencing intimate partner violence. Many participants also discussed their experiences of racism and discrimination within non-Indigenous LGBTIQ + communities.

### Experiences of Violence in Family and Community

Many participants distinguished between the reactions of close parents and siblings and that of older, extended family members such as grandparents. Generally, participants’ experiences were mixed, with some family members accepting and supporting their gender and sexuality diversity, while others found it challenging and exhibited phobic behaviours that impacted on young people’s health and wellbeing. Participants also pinpointed differences between experiences in remote and rural communities versus large urban centres with larger LGBTIQ + populations such as Sydney. Larger urban centres were seen as being more tolerant and open minded to gender and sexuality diversity. Generational differences and the closeness of the relationship also seemed to play a role in the ways in which family reacted to gender and/or sexuality diversity. Family violence in most of these accounts is expressed more through emotional rather than direct physical violence, albeit this meant that young participants felt they could not be themselves or openly express their gender and sexuality diverse in these spaces.

One trans participant, however, spoke about the kinds of explicit threats of physical harm they received from other community members and how they constantly monitored for threats of violence to stay safe within some communities:

… but for mob, like, whether you’re LGBT or not, I think physical safety is a huge presence in your life. Like, you’re always monitoring to protect yourself. And there that is kind of, there that is that kind of violence, like, ever present, and increases if you, with gendered violence, I suppose, or with homophobic violence. And I just actually remembered a circumstance where, like, I was talking to someone about being queer or being trans, and it was an older, an older Aboriginal woman, and she told me that, like her immediate response was, now, I would have been speared. If we were still sticking to cultural traditions, I would have been taken out back and speared. And that actually wasn’t the first time I heard that, I was like “Oh yes,” in my mind, I was like, “Oh yes I, I’ve heard that before”. (Participant 15)

Indigenous LGBTIQSB + peoples have intersectional lived experiences, navigating one community then another, negotiating the different norms and behaviours of their different communities. And even though their experiences in Indigenous communities could be negative, a young queer non-binary transperson found that violence from ‘white settlers’ communities were much worse:

I experienced discrimination pretty much everywhere that isn’t a queer Aboriginal space, which I’m lucky to be exposed to a lot of spaces and I, I tend to prioritise those spaces, or like a queer BIPOC [Black Indigenous People of Color] space. I experience a lot of discrimination from mob, absolutely, I do. And it’s often more, more subtle from mob. But I absolutely have experienced the most violence from not Aboriginal people, from white settlers in my life. I grew up in an area where I would walk down the street and people would throw glass bottles out of their car at me. So, I experienced, you know, extreme violence from white settlers pretty much from the get-go in my life. And I’ve never known mob to be as violent as them, or as discriminatory or as bigoted as white settlers are. And I do genuinely believe that the reasons that mob discriminatory to, to other mob, to queer mob and to trans mob now is the result of the violence white settlers have inflicted on the people that came before us. So, for me, it always comes back to white settler violence. (Participant 15)

A lesbian ciswoman also had difficulties fitting into regional or rural communities because of her sexuality, increasing her feelings of isolation and invisibility:

I think growing up, discovering my sexuality, I feel like being in a regional or rural part of Australia and in a community where I couldn’t visibly see anyone like me stalled the fact – me finding out who I was because if – you don’t know what you don’t know. So, I just felt like everyone else is interested in boys and dating and all those sorts of things and I was just kind of like along for the ride. So, my only – it just – it wasn’t existing. There weren’t conversations – when I would go to the doctors, I never remember seeing like a sign of like rainbow mob, like make sure you get tested, or something like that – never. I felt invisible. This just didn’t exist in my community, and it was a weird thing that I was. (Participant 6)

A gay ciswoman also discussed her experiences of generational and urban-rural differences, often feeling unsafe around older family members:

I’m noticing, when I’m around my family, specifically, older family, my Torres Strait Islander family, it’s not that they’re homophobic, it’s just that they don’t know and they’re just in their times. So, if I was with my peers, I would correct or educate. But when I’m, with my Elders, I just keep quiet and I’m like, this is about me being safe and just, now is not the time. And that’s when I feel really aware of it, especially now, because in Sydney, I can just be like a 100% of myself all the time. But as soon as I get back to Townsville, I’m like, I don’t know, I don’t know how they’re gonna feel about this. (Participant 16)

Not all experiences were negative, however, as a young bisexual ciswoman explains her close family had “queer friends” and were very accepting of her sexuality. However, her family lived in a large urban area with a significant LGBTIQ + community that meant her immediate family at least had connections with the LGBTIQ + community:

I think I’ve always lived in Sydney and my close family were – I came out to them [family] maybe two years ago and they were very – it was fine. I think they have a lot of queer friends. It seemed to be like not an issue but then I never really spoke about it with my extended family (North Queensland) because there are some homophobic people in my family that I don’t really talk about it with. (Participant 3)

This was also the experience of a gay cisman who found that his family had complex reactions, differing from immediate family members to that of extended family:

I mean, we all get on to the rest of my family, but my immediate mum, dad, aunty, cousins, and grandma on dad’s side are all well-receiving, but there is fear from my mum’s mum and my mum’s brothers, my uncles. I don’t know, just ‘cause I guess they’re super traditional in a heteronormative way and have heard them make queer-phobic remarks before. So, I’m reluctant to be myself around them and I regress to that performing masculinity part when they’re near and stuff, so – yeah. (Participant 9)

A young ciswoman also highlighted the different reactions between extended vs. close family, and rural/urban communities, and the attitudinal changes that have shifted in communities over time:

I’ve been pretty fortunate to grow up in a community where it was acceptable. I come out after it was legal to be married … So, I was quite lucky to come out at a time where it was acceptable, with a family that was very accepting as well. But back home in the smaller community of Peak Hill, it’s not very – there’s not many queer people. I’m the only gay person, openly gay person in my family on my mum’s side. So they’re a bit – I don’t talk to most of my family because of that reason. They’re a bit weird about it … So, I’ve got more gay family on my dad’s side. So, when I go back to country, I can’t actually go back to Peak Hill. I said I’m just really uncomfortable there. So, I can’t go home. I go to Dubbo, and I stay in Dubbo. That’s where I feel more comfortable because there’s more gays on dad’s side of the family and they’re more welcoming. Yeah, but that small town of Peak Hill, where there’s 1,400 people in that town, not many, you feel very uncomfortable there, but – yeah, I can’t go back. (Participant 5)

This also was one of the main issues raised by a Brotherboy from a rural town in Queensland:

For me, I’ve lived away from my community to begin with … So, I was not really invested in what the community were saying about me because I knew it wasn’t gonna be good, and it wasn’t until I revisited the community later, after I transitioned, after I’ve moved to Melbourne, I would feel more comfortable in presenting myself as who I am. Yeah, because there was a lot of rejection and there was not a lot of acceptance. So, I went and do my own thing, and then I come back, and they were more willing to accept me for who I am. (Participant 7)

Very few experiences of Intimate Family Violence were mentioned by our participants. Only three people said they had experienced IPV with their partner, two did not go into any detail, only mentioning the fact they had experienced it. Participant 3, for example, said that her feelings of anxiety and depression were, “related to my relationships being bad” and goes on to explain that “I had a relationship that was abusive when I was like 20 or 19”. Participant 4, a gay cisman, discussed the more subtle ways his partner tried to control him, openly loving in private but rejecting him in public, passing as straight and ignoring him while flirting with women in community spaces. This led to the participant feeling disempowered, angry, isolated, and depressed. A young trans participant (Participant 6) provided the most details about intimate partner violence describing a relationship as a teenager that ended in, “a public break-up because there was domestic violence and I had to go to court to get a domestic violence order against her”. Serious concerns arose for this participant as their partner was also Indigenous and this caused a lot of anger directed towards them by their partner’s family and other community members. Their partner’s family, for example, tried to blame them for the situation and for making their daughter a lesbian, while other community members actively took sides. Fortunately, the situation was mediated by the participant’s grandmother, who supported them through the process and acted as an advocate with other families and community: “Like my nana picked me up from school and was like, ‘This is what we’re gonna do.’ We went and run and saw everyone’s families who were bullying me at the time” (Participant 6).

Participants’ experiences of family violence in family and community show that Indigenous LGBTIQ + people experience various forms of family violence within family and community. Many participants reported there were a significant age and generational differences between reactions from parents and siblings and that of older, extended family members such as grandparents. There were also differences in the ways in which urban and rural communities responded, many participants finding it challenging to visit or live in regional or rural communities because of negative reactions to their gender and sexuality diversity. In contrast, others talked about the acceptance they had found in larger urban centres where their families and communities had ties with, or knew people from, local LGBTIQ + communities. Much of these types of behaviors were reported across participants, however, only three participants discussed intimate family violence and we can only speculate as to why this might be the case. Some participants might not have recognized their partners’ behaviours as being of concern or perhaps participants also tried to protect family and community by keeping silent about any instances of intimate family violence (Carlson et al., 2021; Sullivan, 2021). Lusby et al. (2022) also found that many non-Indigenous LGBTIQ + people also found it difficult to identify family violence, many assuming this meant overt physical and/or sexual violence and not “non-physical abusive tactics used to coerce, control or cause fear” (p. 4).

### Racism in non-Indigenous LGBTIQ + Communities

If we consider family violence to encompass not only family but also community, then the intersectionality of Indigenous LGBTIQ + people necessitates that their experiences of community intersect with both Indigenous and LGBTIQ + communities. Participants’ lived experiences within the LGBTIQ + community are consequently important to understand given that most participants actively engaged with and hoped for safe LGBTIQ + spaces where they could be their authentic selves, both Indigenous and LGBTIQ+. The racism and discrimination described here are the acts of individuals in the LGBTIQ + community, even though these behaviours are often embedded and enabled by the policies, practices, and values of white settler institutions.

Indeed, most participants did not speak directly about racism with some also saying that they felt comfortable in non-Indigenous LGBTIQ + spaces. However, participants also said that they did not always disclose that they were Indigenous. Participants spoke about ‘passing’, or what Participant 6 called “passing privilege in that I’m pale, people think I’m white”, which allowed participants to ‘pass’ in the non-Indigenous LGBTIQ + community. Another participant also speculated that there was no racism in the LGBTIQ + community because:

I feel that they [LGBTIQ + community] have also dealt with not belonging. They’ve also dealt with being outsiders, ‘cause being an Aboriginal woman, you deal with of that stuff … And I feel like that the LGBTQ community has been the same – unaccepted, being the outsiders, and whatnot. So, I feel like there’s no racism whatsoever when it comes to being in that community (Participant 5).

A minority of participants spoke of experiencing or witnessing racist behaviours in non-Indigenous LGBTIQ + spaces such as parties, nightclubs, and pubs. These young people reported experiencing emotional and psychological violence in non-Indigenous LGBTIQ + spaces, which has had direct, negative impacts on their wellbeing and their ability to freely go where they wanted. A bisexual ciswoman discusses how she felt when another partygoer interrogated her about her skin color at a queer party:

I think the queer community can be racist in some areas. I think it depends on where the groups that you find. I remember one time I went to this party, it was like a lesbian party, and I was there and then this girl came up to me and she was just like, “Oh, you’re so brave to come here. Your skin is so beautiful,” it was really weird. She was just talking about my skin and making me feel really uncomfortable. She obviously thought it was weird that I was there. (Participant 3)

A gay cisman also felt conflicted about going out to non-Indigenous LGBTIQ + spaces such as nightclubs because of the kinds of racist and misogynist remarks and behaviours he had heard about:

I don’t like going out to the gay clubs in Sydney because you hear so many racist, disgusting things, also very misogynistic things as well, and sometimes it’s not even racist things towards my own people, it’s towards other races, but because I don’t like that, I don’t wanna hear that and, especially from people who are in a community that’s so marginalised against, it just goes beyond my brain, it’s like beating down those who are already beaten enough to the point where they can’t get up, like you’re beating on the even more and you don’t even have a leg to stand on. (Participant 10)

Another participant also spoke of his friends’ experiences of racism in university queer spaces:

So, it hasn’t happened to me, but it’s happened to students for me that have interacted with the queer collective on campus. They actually experience racism in regard to – so, they tried to go to this specific queer space that is on campus for queer people where you can go study, hang out, whatever, and going inside these spaces, they experience racism, discrimination. They were verbally told that they didn’t belong here and blah, blah, blah, all of that, and so – yeah. (Participant 9)

Participants’ lived experiences within the LGBTIQ + community are important to understand as many sought safety in these communities, hoping for a safe haven from wider societal LGBTIQ + phobic values. The interviews did not reveal any instances of institutionalized racism or discrimination although there were instances of individuals within community spaces who were at times overtly racist. Even so, the majority of participants did not speak directly about racism and discrimination in the LGBTIQ + community, some because they were not open about their Indigeneity and others simply said they felt safe in non-Indigenous LGBTIQ + spaces.

## Conclusion

This paper is unique in that is the first discussion of Indigenous LGBTIQ + people’s experiences of family violence in the country now known as Australia. Participants’ stories spoke of family and community violence that focused on intimidation, bullying and threats of violence. These kinds of behaviours were at times aimed at making the young person feel uncomfortable, to feel unwanted, invisible, excluded and even to force them to change their ‘lifestyles’ or at least shape their behaviours so that their gender and sexuality diversity were kept hidden. Even though participants spoke about how they successfully negotiated these events, LGBTIQ + phobic and racist behaviours also caused feelings of isolation, distress, a feeling of invisibility, lack of worth and exclusion.

LGBTIQSB + young people reported significant amounts of violence from communities, some related to their gender and sexuality diversity and other behaviours related to perceptions about their Indigeneity. LGBTIQSB+-phobia in Indigenous families and communities was reported more within extended families, older generations and rural or remote communities. Negative interactions and attitudes from Indigenous families and communities are significant given the importance of culture, family, and community for Indigenous people. These are still central to young Indigenous LGBTIQSB + lives with participants speaking about the heart break of not being able to go home to Country to visit grandparents and Elders in rural and remote communities. Young Indigenous LGBTIQ + young people also spoke about the importance of the LGBTIQ + community in claiming their sexuality and gender identity and the feelings of exclusion and discrimination that sometimes occurred due to their Indigeneity.

There is no data currently available on Indigenous LGBTIQ + experiences of family violence in Australia, so it is difficult to compare our findings with other research. There is a small body of evidence from Canada and the United States that demonstrate that in these countries at least, Indigenous Two Spirit and LGBTIQ + people experience a higher risk of being exposed to family and sexual violence (Evans-Campbell et al., 2006; Lehavot et al., 2010; Ristock et al., 2017; Walters, Simoni & Howarth, 2001). However, no such information is available in Australia and given the importance of place and culture, and the unique history and progression of invasion and colonisation, it would be difficult to make too many comparisons. Hopefully, though, this article will initiate a process of opening up discussions around family violence, reinforcing and adding to discussions already underway around LGBTIQ + experiences, but further decentering heteronormative and cisgendered framings of the topic by adding the unique voices and experiences of Indigenous LGBTIQ + people. Unique to Indigenous LGBTIQ + young people was the importance of returning to Country, connecting with culture, family, and community in addition to securing safe spaces where they could just relax and be themselves in the non-Indigenous LGBTIQ + community. This strong connection to culture and community of origin is quite unique and the connections between Indigeneity, culture, and sexuality and/or gender diversity certainly need further investigation. Indeed, this paper has not even scratched the surface on this topic and there is a clear and urgent need for future research to improve and refine our knowledge about Indigenous LGBTIQ + people’s intersectional experiences of navigating and living in Indigenous and non-Indigenous communities. Any future research initiative needs to be led, designed and implemented by Indigenous LGBTIQ + researchers or at the very least, codesigned and led by Indigenous and/or LGBTIQ + research teams.

## Footnotes

1 Please note that in this article we use LGBTIQSB + for Indigenous youth only. For non-Indigenous LGBTIQ + people, we use the LGBTIQ + acronym as they do not include Sistergirls and Brotherboys. These latter terms are not used within all Indigenous communities or by all Indigenous trans people (Sullivan, 2018). Sistergirls are often translated as “transwomen” and Brotherboys as “transmen”, however, but this is not an accurate depiction as it fails to include Indigenous understandings of gender diversity (Riggs & Toone, 2017).
